# The Effect of Sports Performance Entrepreneurial Project by Entrepreneurial Spirit

**DOI:** 10.3389/fpsyg.2022.914388

**Published:** 2022-06-29

**Authors:** Shuxiong Song

**Affiliations:** Physical Education Department, Anhui Science and Technology University, Fengyang, China

**Keywords:** entrepreneurial enterprise, entrepreneurship, sports performance entrepreneurship, performance, entrepreneurial spirit

## Abstract

This study aims to analyze the effect of entrepreneurship on the growth of entrepreneurial enterprise projects, so that entrepreneurial projects can improve entrepreneurial performance, promote social and economic development, and improve resource allocation. Firstly, entrepreneurship is explored to analyze the role in the growth of entrepreneurial enterprise projects. The entrepreneurial essence requirements based on entrepreneurship provide a theoretical basis for sports performance entrepreneurship. Secondly, the idea and process of sports performance entrepreneurship are described. A growth model of sports performance entrepreneurship based on entrepreneurship is established. Finally, the entrepreneurs who started sports performances are considered to investigate the enterprises participating in a sports competition performance. The influence of six elements of entrepreneurship on the progress and performance of sports performance entrepreneurial projects is analyzed. The results show that the spirit of risk-taking, need for achievement, and professionalism in entrepreneurship have the highest consistency, all above 0.90. It shows that entrepreneurship can promote the sports performance entrepreneurship project, and the subjects have a high entrepreneurial spirit. The adventurous spirit has more than half of the coverage of sports performance entrepreneurial projects. The coverage rate of knowledge literacy, innovative spirit, advanced action, achievement needs, and professionalism in sports performance entrepreneurial projects are all about 0.43. Innovation, risk-taking, and creativity can boost the performance of sports performance entrepreneurial projects. Enterprise size has little influence on sports performance. These conclusions have reference significance for the impact of sports entrepreneurial project performance.

## Introduction

In recent years, investment in China's sports industry has shifted from government-led to market. The rapidly growing demand for sports consumption has attracted social capital from all walks of life to rush into the sports industry. The emerging new sports formats and models are an important driving force for activating the development potential of the sports economy (Zhuo et al., 2020). Sports industry innovation should be deconstructed from three dimensions: industrial integration, sports scene, and industrial organization (González-Serrano et al., 2020). The State Council of the People's Republic of China has issued policy documents concerning the sports industry and the fitness and leisure industry. In order to further optimize the structure of the sports industry, accelerate the development of the sports performance industry, improve the standard level of industrial development, and promote the healthy and orderly development of the industry, the document issued guidance opinions on accelerating the development of sports performance industry from the national level (Jaros and Tan, 2020). “Vitality of the market comes from people, especially from entrepreneurs, from the entrepreneurial spirit.” Since the 18th National Congress of the Communist Party of China, General Secretary Xi Jinping has attached great importance to the important role of entrepreneurial groups in national development. The chairman has repeatedly emphasized the need to carry forward entrepreneurship (Mertens and Thiemann, 2019). For a long time, entrepreneurship has been defined in terms of business, management, operation, organization, leadership, and its own essential characteristics (Parente et al., 2021). After entering the 20th century, the abstract concept of entrepreneurship has been defined in various fields of behavior, psychology, and sociology (Rogoza et al., 2018). In today's Western countries, entrepreneurship has been transformed and served in various social organization work, managing social work (Peerally et al., 2019). In the 2010s, the Internet, especially social networks and media sites, boosted social entrepreneurship (Shen et al., 2019). These sites allow social entrepreneurs to reach out to many people who are not geographically close but share the same goals and encourage them to collaborate online, learn about issues, and spread information about the organization's activities (Feng and Chen, 2020).

In order to meet the needs of consumers for sports competitions, the organizers of sports performances have carried out a series of economic activities to provide various sports performance products to the market (Ye and Chen, 2021). With the rapid development and rise of technologies such as the Internet and big data, more and more industries and the Internet are showing a trend of integration (Zhang, 2021). The combination of network media, big data technology, and sports can open the development space of the sports industry. The sports competition performance industry is the core component of the sports industry. Its integration with the Internet has begun to take shape (Wang and Zeng, 2020). Looking at the companies and organizers of foreign sports competitions, sports clubs pay attention to social media and mobile technology (Happ et al., 2021). For example, Wimbledon Championships uses new technologies and social platforms for promotion and marketing. Wimbledon, for example, is using new technology and social media to promote its events. The Open has far more followers on Twitter, Facebook and Instagram than the US, French and Australian Opens. In 2014, mentions, page views and unique visitors to the platform reached 6, 63 and 17.1 million, respectively (Subramanian et al., 2020) (Subramanian et al., 2020). Tencent Sports cooperated with the National Basketball Association (NBA) to realize the online and offline interactive entertainment mode. The creation of derivative businesses such as Tencent QQ, barrage, and interactive entertainment not only enhances the influence of Tencent Sports but also plays a positive role in the promotion and marketing of related sports events and the increase of sports fan groups (Xue et al., 2020). The needs for rapid social and economic development in two-thirds of the world's poor areas, needs in large cities, needs in the environment, and needs in education and health care. These needs are all kinds of opportunities for enterprises and managers in social innovation and all kinds of opportunities that entrepreneurs will face in the future (Liu et al., 2020). Additionally, the huge user group also poses new challenges and requirements for entrepreneurs to manage knowledge, skills, and performance (Makhloufi et al., 2022).

Firstly, entrepreneurship is explored. The entrepreneurial nature of entrepreneurship requires a theoretical basis for sports performance entrepreneurship. Secondly, the idea and process of sports performance entrepreneurship are described. A growth model of sports performance entrepreneurship based on entrepreneurship is established. Finally, the entrepreneur of the sports performance business is used as the object. Participating companies are investigated in sports competitions. The influence of the six elements of entrepreneurship on the progress and performance of sports performance entrepreneurial projects is analyzed separately. This study can be used to study the influence of entrepreneurship on the performance of sports performance entrepreneurship projects. Innovation not only lies in perfecting and enriching entrepreneurial theories but also in deepening the growth of entrepreneurial projects. Additionally, the questionnaire is used to study the impact of sports performance entrepreneurship projects, opening up a new perspective on the effect of entrepreneurship projects.

## Literature Review

Since the 21st century, according to the research on the world economic situation, science and technology have brought large-scale changes to people's production and life. The world economic situation has also begun to change from management-oriented to entrepreneurial. Habib et al. (2020) analyzed the impact of entrepreneurship on entrepreneurial orientation and the process of enterprise development. They found that entrepreneurship promotes the positive development of entrepreneurial orientation. Entrepreneurial orientation positively promotes performance. In 1983, after the concept of entrepreneurship was put forward, researchers in the direction of management attracted more attention to this aspect. From the management perspective, Hanckel et al. (2021) explored the configuration perspective and the Qualitative Comparative Analysis (QCA) method. Al-Awlaqi et al. (2021) analyzed the impact of entrepreneurship on enterprise growth. The study found the combined effect mechanism of entrepreneurship elements on the growth of entrepreneurial enterprises. In view of the social research status of sports performance majors, Martinez et al. (2020) demonstrated the necessity of adding sports performance undergraduate majors in universities and researching the development prospects and feasibility of sports dance direction. Hsia et al. (2022) proposed a method for cultivating students' practical ability for sports performance majors, which improved students' practical ability. O'Connor and Penney (2021) studied the existing problems in the training of sports performance talents in schools. At this stage, the sports performance major is in the development stage. Still, the quality of talent training in sports performance is not high, and there is incompatibility with social needs, etc.

Based on the improvement of sports talent quality, the impact of entrepreneurship on sports performance is analyzed. This study not only analyzes the relationship between entrepreneurship and performance but also guides the entrepreneurship of sports performance majors in schools.

## Method

### Exploration of Entrepreneurship

Entrepreneurship is a collection of the special skills of entrepreneurs, which refers to the expression of the comprehensive skills of entrepreneurs to lead the construction, operation, and management of enterprises. It is an intangible factor of production (Doern et al., 2019). Entrepreneurship is an important factor in the long-term development of a company. The components of entrepreneurship are shown in Table 1.

**Table 1:** Components of entrepreneurship.

**Composition factor**	**Content**	**Essential features**
Innovation	From product innovation to technological innovation, market innovation, and organizational form innovation	The soul of entrepreneurship
Adventure	Have the courage to take risks and take risks	Entrepreneurial nature
Cooperate	Implement collective action in major decisions	The essence of entrepreneurship
Dedicated	Live for business	Power of entrepreneurship
Learn	The whole enterprise is continuous learning, full learning, team learning, lifelong learning	The key to entrepreneurship
Perseverance	Perseverance and continuous innovation	The essence of entrepreneurship
Integrity	The market economy is an economy ruled by law, an economy of credit, and an economy of integrity.	The cornerstone of entrepreneurship

**Figure 1:**
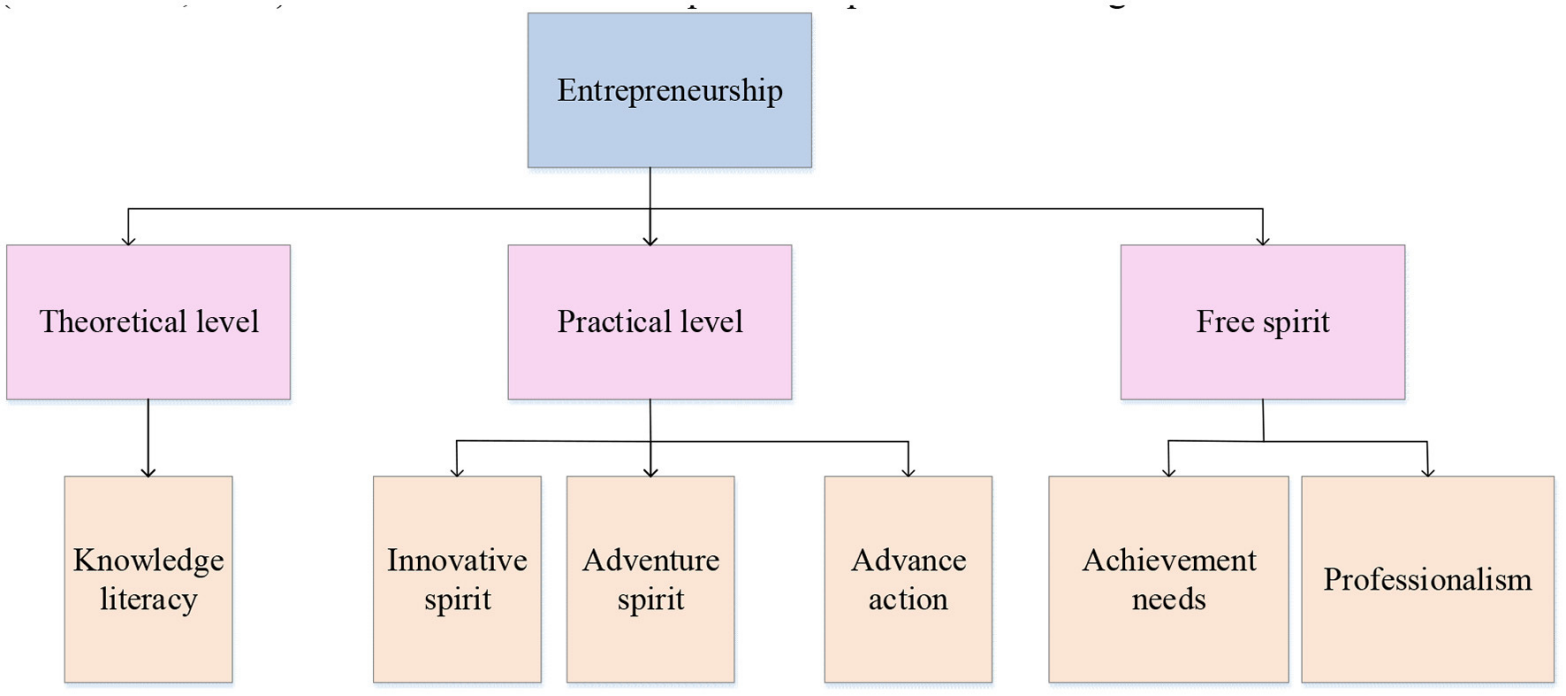
The six elements of entrepreneurship.

In Table 1, the core point of entrepreneurship is innovation. Entrepreneurial leadership is equivalent to management (García et al., 2022). This is consistent with Peter Ferdinand Drucker's view that “the core content of business management is the entrepreneur's economic risk-taking behavior. The business is the organization in which the entrepreneur works” (Bao and Wang, 2022). Social entrepreneurship is an approach by which individuals, groups, start-ups, or entrepreneurs develop, invest in, and implement solutions for social, cultural, or environmental issues (Bansal et al., 2019). This concept can be broadly applied to organizations of all sizes, purposes, and beliefs. Profitable entrepreneurs typically measure performance by business metrics such as profits, revenue, and share price increases. However, social entrepreneurs are either not-for-profit or combine for-profit goals with creating positive “social returns” (Wach et al., 2020). The six elements of entrepreneurship are shown in Figure 1.

**Figure 2:**
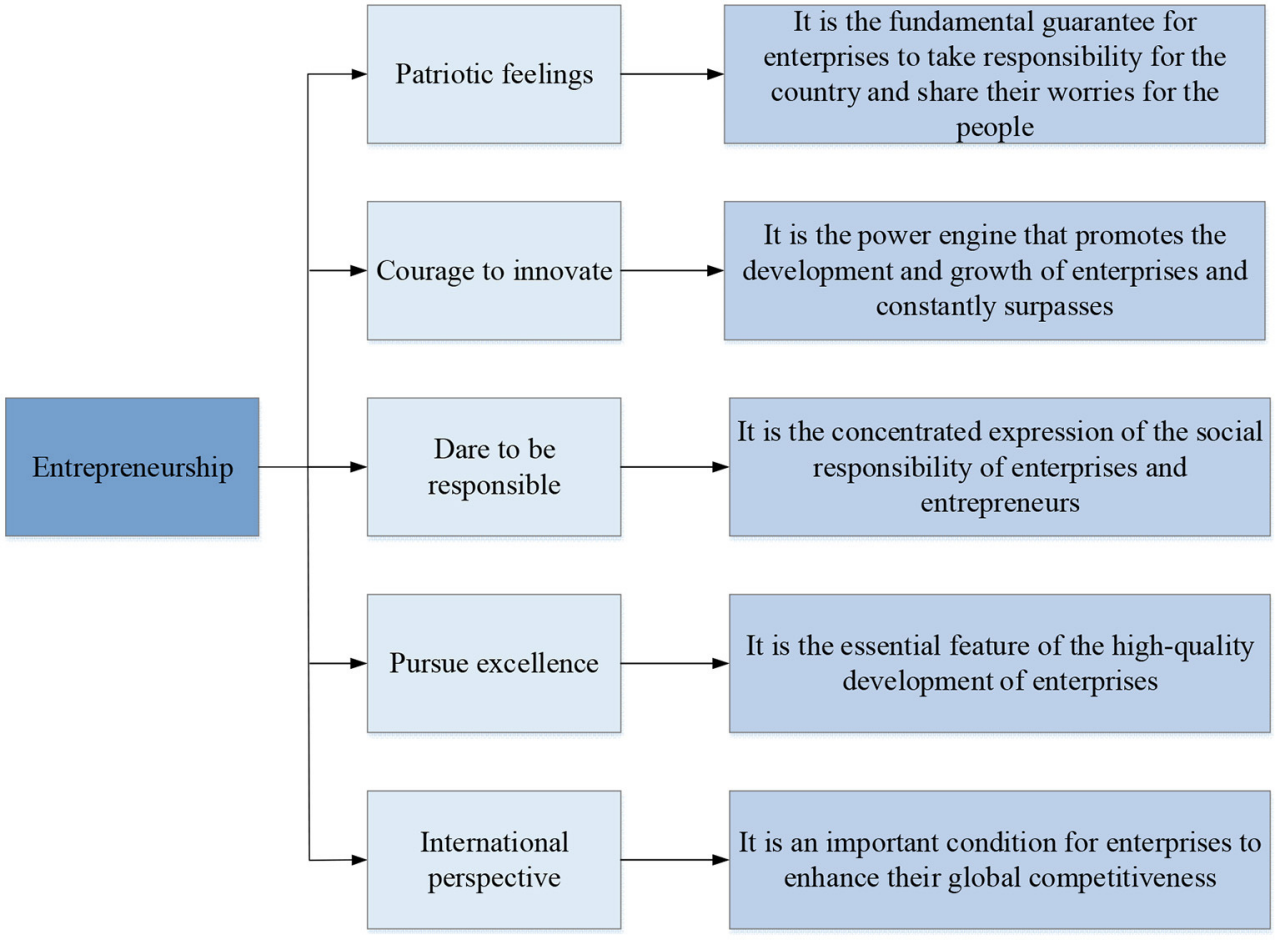
Essential requirements for entrepreneurship based on entrepreneurial spirit.

In Figure 1, the elements of entrepreneurial success are used as the starting point. This part expounds on three dimensions that influence the success of entrepreneurship, namely, theoretical perspective, practical perspective, and automation level, which mainly include knowledge accomplishment, innovative spirit, adventurous spirit, advanced action, achievement demand, and professional spirit (Shakeel et al., 2020). The main body of the market is the power carrier of the economy. Enterprises are the most important market entities, major participants in economic activities, major providers of employment opportunities, and major promoters of technological progress and play a very important role in economic development (Tran, 2019). The essential requirements of entrepreneurship based on entrepreneurship are shown in Figure 2.

In Figure 2, patriotism is the essential requirement of entrepreneurship in the new era, and patriotism is the duty and legal bottom line of every citizen. The entrepreneur who is the main person in charge of the enterprise is not only a natural person, a citizen but also the legal representative of a legal person organization of a market entity (Obschonka and Audretsch, 2020). Dare to innovate is the essence of entrepreneurship in the new era. Innovation reflects the wisdom and efforts of enterprise managers. It is the key factor for enterprises to achieve breakthrough development and catch up with competitors. Entrepreneurs are the backbone of developing an innovative economy and realizing a strong manufacturing country in the new era (Wanqing et al., 2021). It is the special mission of entrepreneurship in the new era. A responsible manager is also an entrepreneur with a sense of social responsibility. Solving employment problems is the social responsibility of enterprises, developing innovative products is the professional responsibility, and sharing experience and results are the industry's responsibility. The pursuit of excellence is an important manifestation of the entrepreneurial spirit in the new era, and the pursuit of excellence is to promote the spirit of craftsmen. Its essential characteristics lie in the attitude and dedication of dedication, concentration, and excellence in their own work. An international perspective is an era requirement for entrepreneurship in the new era. Entrepreneurs must actively respond to changes in the international situation, actively participate in international market competition, design enterprise development strategies and competition strategies from globalization, and develop and consolidate international markets with high-quality products and services (Dung and Tri, 2021).

### Sports Entrepreneurship Performance Project Based on Entrepreneurship

**Figure 3:**
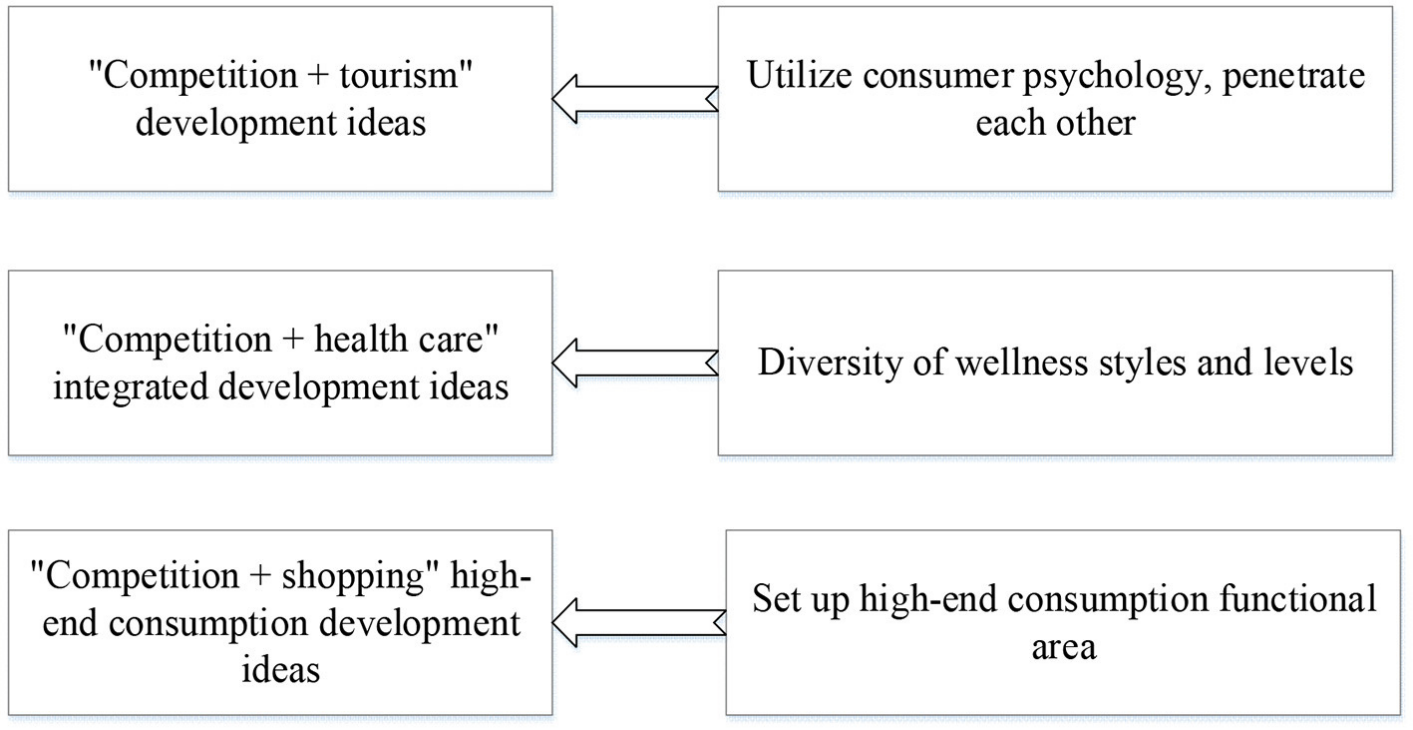
Sports performance entrepreneurship ideas.

The sports performance industry is an important part of sports. The development of the sports performance industry is of great significance to tap and release consumption potential, ensure and improve people's livelihood, and create new momentum for economic growth (Arenas-Jal et al., 2020). The sports performance industry faces development trends such as small industrial scale, unreasonable structure, and unbalanced regional development (Kan and Lam, 2021). According to the international classification, sports can be divided into two categories: participatory and spectator sports. Participatory sports mainly refer to fitness and leisure, and spectator sports mainly refer to performances (Wei and Zhao, 2020). As a result, the sports industry can be divided into two formats formed around fitness and leisure and performance activities. The sports performance entrepreneurial idea is shown in Figure 3.

**Figure 4:**
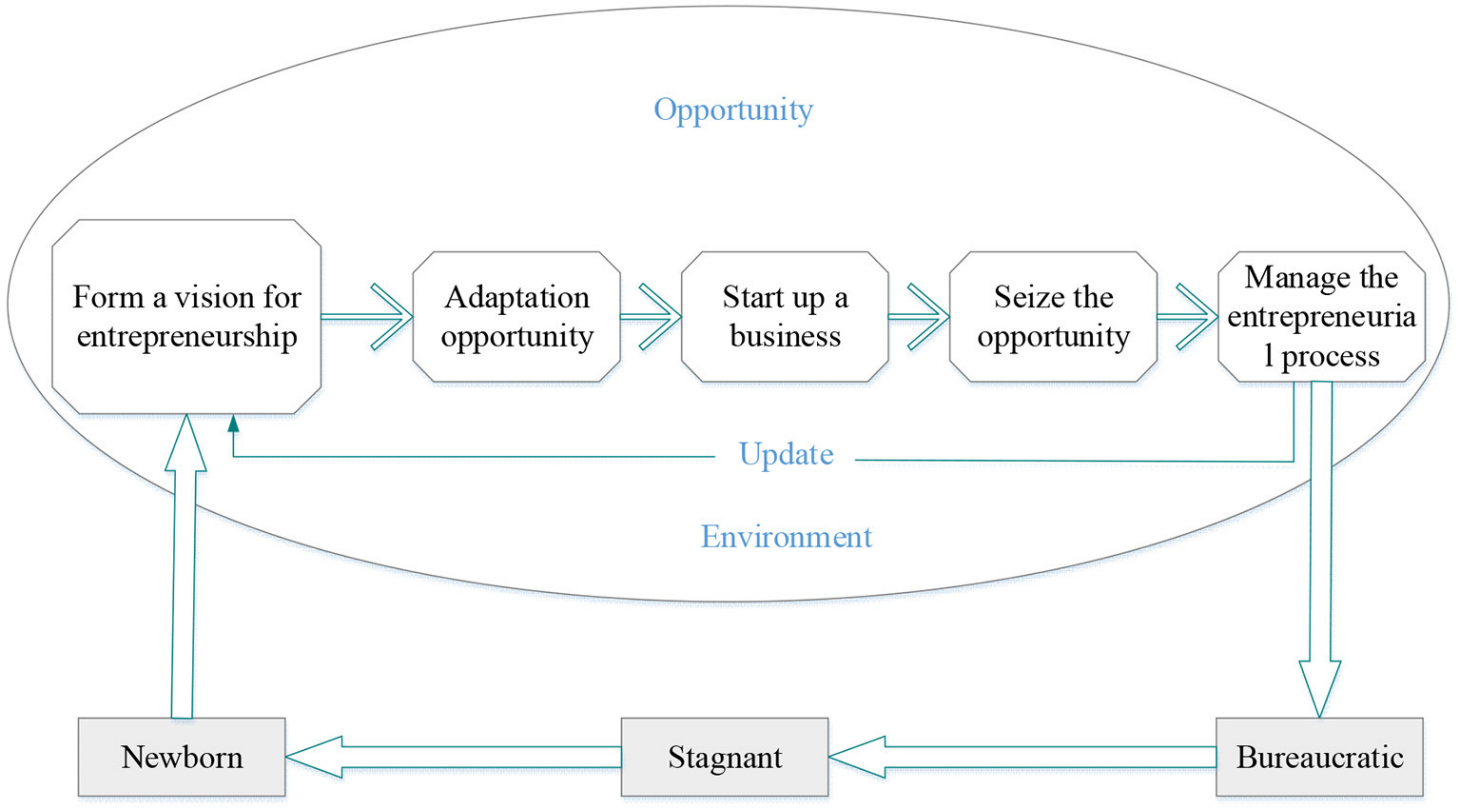
Entrepreneurial process approach.

In Figure 3, there are three different approaches to sports entrepreneurship. Growth-oriented innovative and entrepreneurial enterprises often represent new technologies, industries, formats, and applications and are full of vitality. However, many growth-oriented innovative and entrepreneurial enterprises are relatively weak and urgently need strong support from the capital market (Zheng and Liu, 2020). When an organization grows and becomes larger, the method of business operation will change accordingly, and elements such as structure and process control will become bureaucratic. At this time, the entrepreneurial spirit must be instilled and extended so that the entrepreneurial spirit can be preserved. The entrepreneurial process approach is shown in Figure 4.

**Figure 5:**
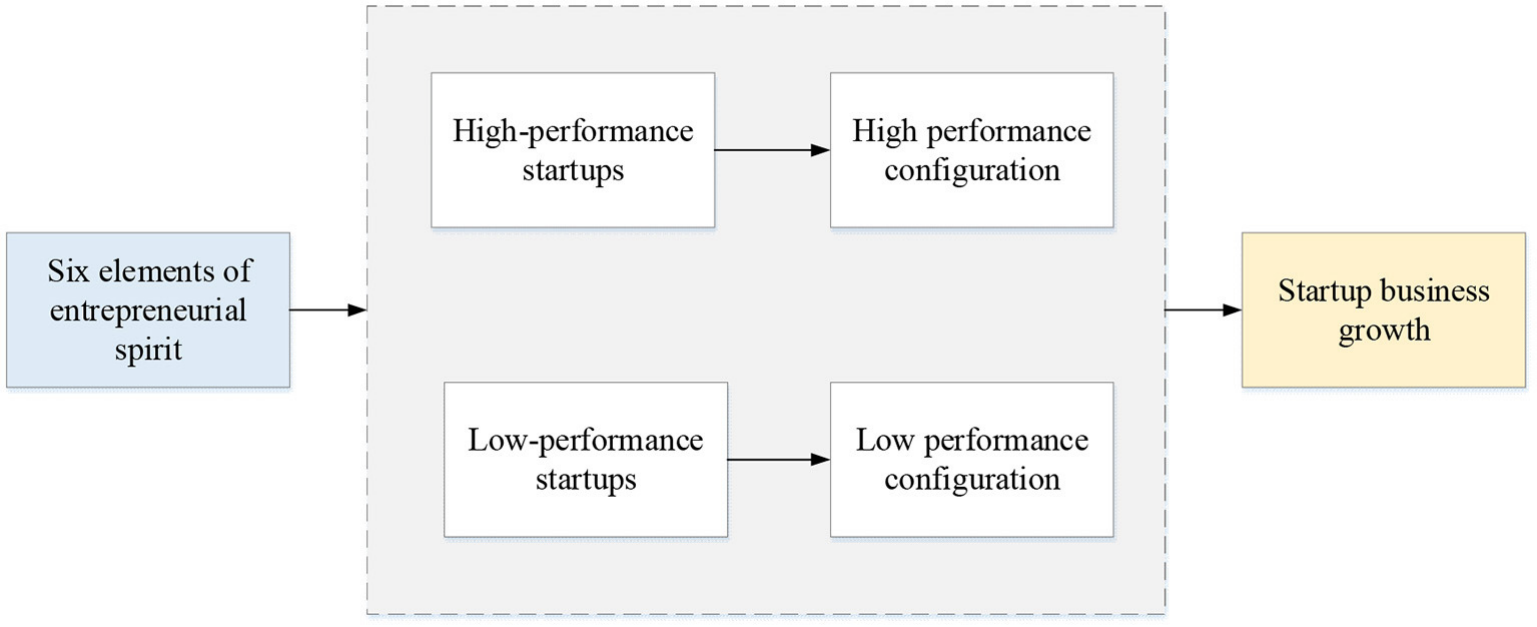
Growth model of sports performance entrepreneurship based on entrepreneurship.

In Figure 4, the entrepreneurial process approach has persistent uncertainty in the operational transition of entrepreneurial organizations. Navigating this uncertainty requires paying close attention to capturing opportunities. Opportunities arise anywhere in the context of business operations and organizations. Organizations must change dynamically to seize opportunities (Dileo and García Pereiro, 2019). The sports performance entrepreneurial growth model based on entrepreneurship is shown in Figure 5.

In Figure 5, the relationship between six elements of entrepreneurship and enterprise growth is studied. The effect of entrepreneurship elements on high performance and low performance of enterprise growth is analyzed.

### Questionnaire Survey Method for Sports Performance Entrepreneurial Projects

The 11 categories of China's sports industry can be summarized as the industrial system of the two major industrial chains of the sports leisure fitness industry and the competitive performance industry. Entrepreneurs of sports performance business are used as objects. Businesses involved in sports competition performances are investigated. For example, Tencent Sports and NBA officials have become in-depth cooperation partners. Tencent Sports reports the latest NBA finals, presents massive information and conducts comprehensive reports to realize the online and offline communication mode. However, while the influence of Tencent Sports has increased, the right to live broadcasts of sports events has been damaged by the infringement of a large number of illegal online platforms. Those platforms have affected the healthy and rapid development of the competitive performance industry. Compared with the China Basketball Association (CBA) league, the NBA league is technically more comprehensive and digital. The NBA data analysis awareness is ahead, and the CBA game data and query system need to be improved. The sports competition and performing arts industry should be deeply integrated with new online media to realize intelligent event services and venue operations. Phoenix, Sina, Sohu, Tencent, and Netease are five comprehensive portal websites that use Internet technology to broadcast sports events. Users can watch live matches and replays anytime, anywhere. These sites provide users with important platforms for sporting event information and competitions. Alibaba, JD, Tmall, and other comprehensive e-commerce platforms, based on the continuous integration of the clothing and props industry and new media, create an online clothing and props market to meet the needs of sports consumers for quick shopping.

The impact of the six elements of entrepreneurship on the progress and performance of sports performance entrepreneurial projects is further analyzed, and the results are released through the official website of sports competitions and public accounts. Two hundred sixteen questionnaires are distributed online, and 193 questionnaires are recovered. Coverage is a measure of test integrity, a measure of test effectiveness, and is used for reliability and stability and performance evaluation. Test coverage is a measure of test completeness. The consistency rate and coverage rate of the questionnaire for the research questions are calculated, as shown in Equations 1 and 2:

(1) $$\begin{array}{r} Consistency\left(X_{i}\leq Y_{i}\right)=\sum \left[\min\left(X_{i}\leq Y_{i}\right)/\sum \left(X_{i}\right)\right] \end{array}$$

(2) $$\begin{array}{r} Coverage\left(X_{i}\leq Y_{i}\right)=\sum \left[\min\left(X_{i}\leq Y_{i}\right)/\sum \left(Y_{i}\right)\right] \end{array}$$

*X*_*i*_ is the calibration value of the conditional elements of the questionnaire, and *Y*_*i*_ is the calibration value of the explanatory power of the questionnaire to the research question. The reliability test of the questionnaire is shown in Equation 3:


(3) $$\alpha =\frac{N}{N-1}(1-\frac{\sum S_{i}^{2}}{S_{x}^{2}})$$

α is Cronbach's α, *N* is the number of questions, $S_{i}^{2}$ is the variance of the scores of all subjects on item *i* (Hayes and Coutts, 2020), and $S_{x}^{2}$ is the variance of the total scores of all subjects in the questionnaire. Composite reliability (CR) is calculated as shown in Equation 4:

(4) $$\begin{array}{r} \rho =var\left(\sum_{i-1}^{N}\lambda_{i}\xi \right)/\left[var\left(\sum_{i=1}^{N}\lambda_{i}\xi \right)+\left(\sum_{i=1}^{N}\delta_{i}\right)\right] \end{array}$$

In Equation 4, λ_*i*_ is the loading of different topics on the latent variable, ξ is the latent variable, and δ_*i*_ is the standardized factor loading. The average variance extracted (AVE) is constructed, and measures share the average variance as shown in Equation 5:

(5) $$\begin{array}{r} AVE=\sum {\lambda_{i}}^{2}/\left(\sum {\lambda_{i}}^{2}+\sum var\left(\xi \right)\right) \end{array}$$

## Results and Discussion

### Analysis of the Reliability and Validity of the Questionnaire

**Figure 6:**
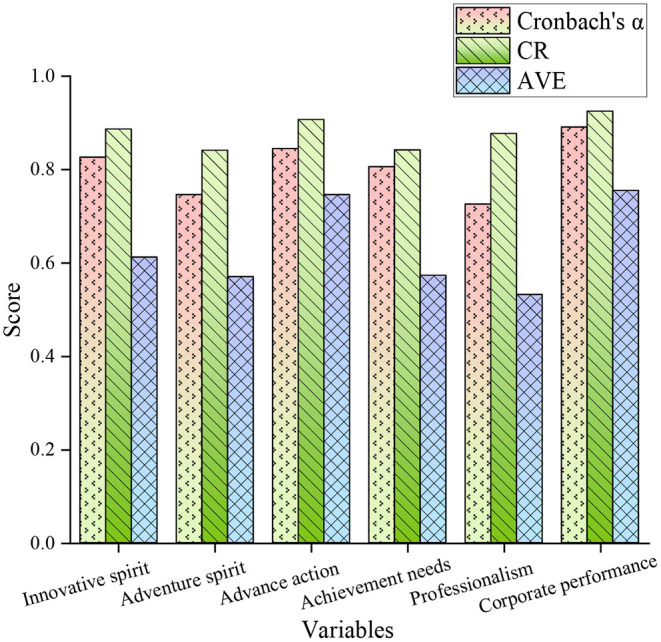
Analysis of the reliability and validity of the questionnaire.

The questionnaire innovation spirit, risk-taking spirit, advanced action, achievement needs, professionalism, and enterprise performance are selected as the influencing factors for the reliability and validity analysis of the questionnaire, as shown in Figure 6.

In Figure 6, the average Cronbach's alpha of all impact factors is 0.81, and the average CR is 0.88. The data shows that entrepreneurship has good reliability for the questionnaire of sports performance entrepreneurship projects. The mean-variance of all influencing factors is 0.63, indicating that the influencing factors had good convergent validity in the questionnaire.

### Analysis of the Performance Consistency and Coverage of Sports Performance Entrepreneurship Projects by Entrepreneurship Elements

**Figure 7:**
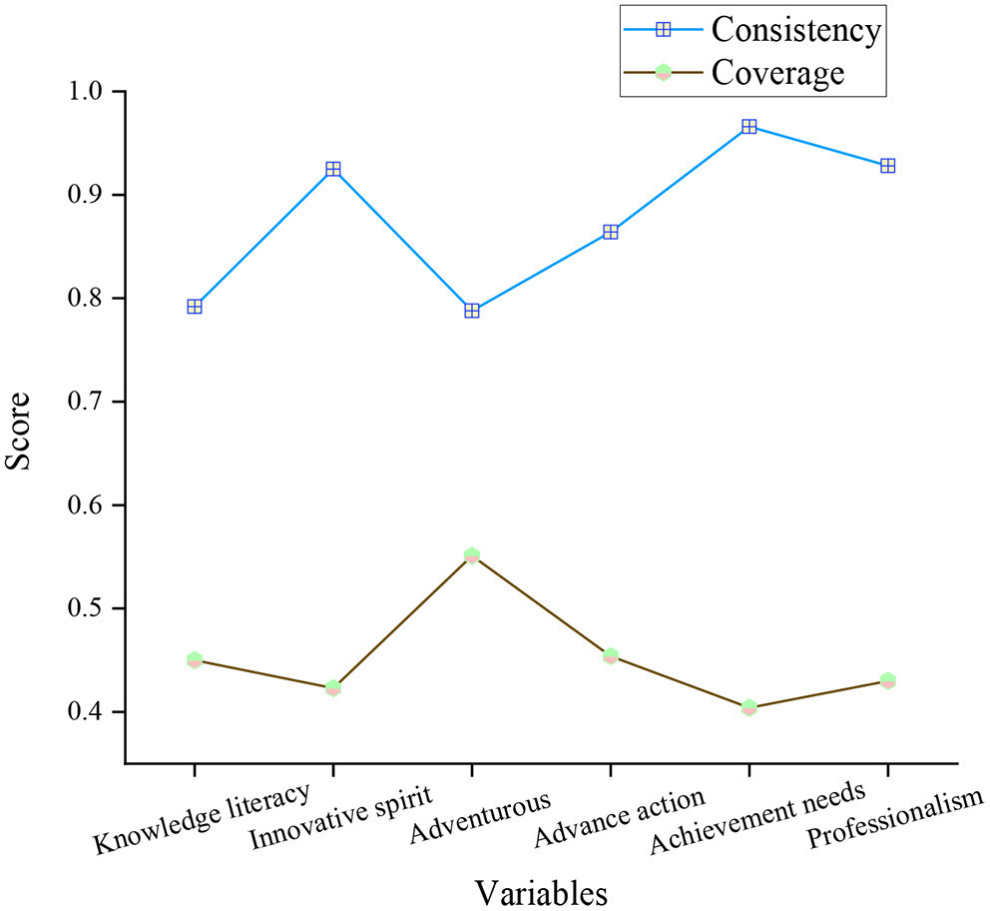
Analysis of the performance consistency and coverage of sports performance entrepreneurship projects by entrepreneurship elements.

The necessity of the performance of sports performance entrepreneurship projects is tested based on the six elements of entrepreneurship. Through the consistency and coverage test, the analysis of the performance consistency and coverage of entrepreneurship elements on sports performance entrepreneurship projects is shown in Figure 7.

In Figure 7, the average consistency of the six elements of entrepreneurship in sports performance entrepreneurship projects is 0.87. The spirit of adventure, need for achievement, and professionalism has the highest consistency, all above 0.90. The data indicate that entrepreneurship promotes sports performance entrepreneurship projects. The subjects have a high entrepreneurial spirit. The spirit of adventure has more than half of the coverage in sports performance entrepreneurship projects. The coverage rate of knowledge literacy, innovative spirit, advanced action, achievement needs, and professionalism in sports performance entrepreneurship projects is about 0.43.

### Analysis of the Impact of Entrepreneurship on the Performance of Entrepreneurial Sports Projects

**Figure 8:**
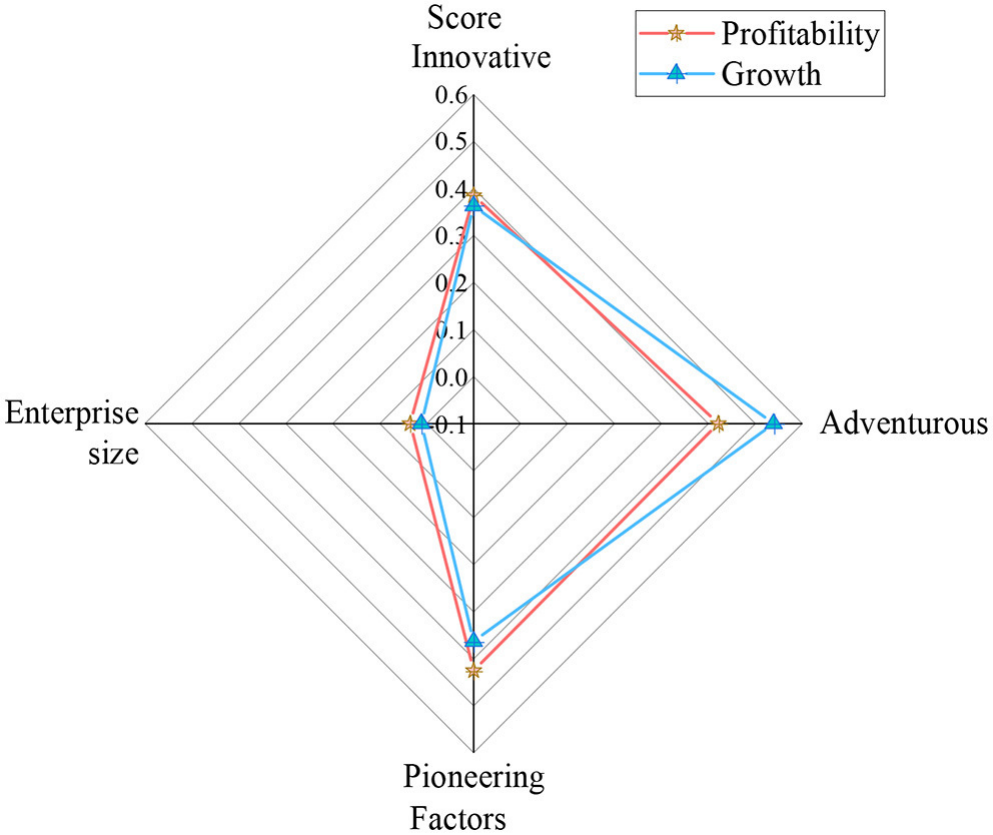
Analysis of the impact of entrepreneurship on the performance of sports performance entrepreneurial projects.

According to sports performance entrepreneurial projects, sports performance entrepreneurial enterprises are divided into profitability and growth. Entrepreneurship innovation, risk-taking, and pioneering are analyzed. The scale of sports performance entrepreneurial enterprises is analyzed as an impact factor. The analysis of the impact of entrepreneurship on the performance of sports performance entrepreneurial projects is shown in Figure 8.

In Figure 8, the impact of innovation on for-profit sports performance companies scored 0.38, and growth companies scored 0.36. Risk-taking has an impact score of 0.42 for for-profit sports performance businesses and a score of 0.54 for growth businesses. The impact score of ground-breaking on for-profit sports performance businesses is 0.43, and the score for growing businesses is 0.36. Enterprise-scale scores 0.04 in sports performance profitable enterprises and 0.01 in growing enterprises. To sum up, innovation, adventure, and entrepreneurship innovation can promote the performance of sports performance entrepreneurship projects. Additionally, the size of the enterprise has little effect on the performance of sports performance entrepreneurship projects.

### Discussion of Results

A questionnaire is used to analyze the impact of entrepreneurship on sports performance entrepreneurial projects. Through research analysis, the reliability of this questionnaire is good. Based on the influence of the six elements of entrepreneurship in sports performance entrepreneurship projects, the results show that entrepreneurship has a promoting effect on sports performance entrepreneurship projects. When Abbas et al. studied the role of entrepreneurship on enterprise growth, they found that lack of entrepreneurship would have a negative impact on enterprise growth (Abbas et al., 2019). Their conclusions are consistent with the results. In addition, the impact of entrepreneurship on the performance of sports performance entrepreneurial projects is analyzed. When Lerman et al. studied the influence of entrepreneurial nerve and entrepreneurial performance, they found that entrepreneurship is conducive to improving entrepreneurial performance (Lerman et al., 2021). The impact of enterprise-scale on the performance of sports performance entrepreneurial projects is not great. Still, entrepreneurship has an innovative, adventurous, and pioneering role in promoting sports performance entrepreneurial projects. Entrepreneurship has a far-reaching impact on entrepreneurial projects, and it also has certain improvements to entrepreneurial projects, which can improve the performance of entrepreneurial projects under certain conditions.

### Suggestions for the Development of Sports Performance Entrepreneurship

From “fragmentation” thinking to “integration,” suggestions are put forward for the development of the sports performance industry to put forward guiding opinions and a good entrepreneurial atmosphere for sports performance entrepreneurship, and plan and lead the development of the sports performance industry from five aspects:

1. Propose quantifiable development goals for the sports performance industry. By 2025, the total scale of the sports performance industry will reach 2 trillion yuan. China will build several influential sports event cities and sports performance industry clusters, launch 100 high-profile sports events, create 100 sports performance brands with independent intellectual property rights, and cultivate a group of strong sports performances. A competitive sports performance enterprise will be cultivated in the market. These enterprises will become an important force in promoting the sustainable development of the economy and society.
2. Enrich the competition activities and improve the competition system. By vigorously developing professional leagues and actively cultivating sports events, China will establish a rich and diverse sports performance industry system and cultivate a stable audience and project culture. This measure can promote the interaction and integration of sports and cultural performances and create a sports performance brand project with national characteristics.
3. Strengthen market entities and optimize the market environment. The sports department will encourage sports performance enterprises with their own brands, innovation ability, and competitive strength. The government will vigorously promote the reform of the commercial system and provide a good access environment for the sports performance industry. The state will strengthen innovation and entrepreneurship education services in the sports industry and innovate the talent training mechanism. China will attach importance to and encourage the application of high-tech in the sports performance industry and give full play to the role of various intermediary and consulting agencies. The government will guide the public to establish a more active concept of sports consumption, increase the willingness for sports consumption, and strictly investigate and punish illegal activities such as the resale of event tickets to protect the legitimate rights and interests of consumers effectively. The sports department will improve consumption conditions, implement an integrated model of stadium design, construction, operation, and management, organically combine the needs of event functions with comprehensive post-match utilization, and guide sports performance enterprises to participate in the operation of stadiums. These measures will revitalize venue resources.
4. Optimize industrial layout and strengthen platform construction. China will improve the industrial chain and form a service system for the sports performance industry with supporting industries, industrial linkages, and efficient operation. The sports department will improve the industrial standards and promote the construction of the sports performance industry-standard system following the principles of overall planning and step-by-step implementation. China will strengthen the legal protection of the ownership, transfer, and income of sports events-related rights and promote the fair, just, and open transfer of resources with trading conditions, such as the right to hold events, broadcast events, and the right to transfer athletes.
5. Strengthen coordination and cooperation, and strengthen financial security. China will continue to promote the reform of the sports event approval system. For the approval items related to sports events, the relevant departments shall not require the event organizers to submit the approval materials from the sports department. The government will establish an industry credit system covering performance organizers, practitioners, and contestants and implement joint punishments following relevant regulations. The sports department will improve the relevant investment mechanism, guide social forces to participate, encourage social capital to set up industrial development investment funds, and further expand the financing channels of sports performance institutions.

## Conclusion

This study studies the effect of sports performance entrepreneurship projects based on entrepreneurship theory. The results show that the average consistency of the six elements of entrepreneurship in sports performance entrepreneurship projects is 0.87. The spirit of adventure, need for achievement, and professionalism has the highest consistency, all above 0.90. This shows that the entrepreneurial spirit promotes the sports performance entrepreneurship project, and the subjects have a high entrepreneurial spirit. The spirit of adventure has more than half of the coverage in sports performance entrepreneurship projects. The coverage rate of knowledge literacy, innovative spirit, advanced action, achievement needs, and professionalism in sports performance entrepreneurship projects is about 0.43. The score of innovation and novelty's impact on profitable sports performance companies is 0.38, and the score of growth companies is 0.36. Risk-taking has an impact score of 0.42 for for-profit sports performance businesses and a score of 0.54 for growth businesses. The impact score of ground-breaking on for-profit sports performance businesses is 0.43, and the score for growing businesses is 0.36. Enterprise-scale scores 0.04 in sports performance profitable enterprises and 0.01 in growing enterprises. These conclusions have reference significance for the impact of entrepreneurship on the performance of sports performance entrepreneurial projects. However, due to certain errors in the research results, there is a lack of methodological research on the effect of sports performance entrepreneurship projects. It is hoped that future research can make up for it.

## Data Availability Statement

The raw data supporting the conclusions of this article will be made available by the authors, without undue reservation.

## Ethics Statement

The studies involving human participants were reviewed and approved by Anhui Science and Technology University Ethics Committee. The patients/participants provided their written informed consent to participate in this study. Written informed consent was obtained from the individual(s) for the publication of any potentially identifiable images or data included in this article.

## Author Contributions

The author confirms being the sole contributor of this work and has approved it for publication.

## Conflict of Interest

The author declares that the research was conducted in the absence of any commercial or financial relationships that could be construed as a potential conflict of interest.

## Publisher's Note

All claims expressed in this article are solely those of the authors and do not necessarily represent those of their affiliated organizations, or those of the publisher, the editors and the reviewers. Any product that may be evaluated in this article, or claim that may be made by its manufacturer, is not guaranteed or endorsed by the publisher.

## Appendix

The influence of entrepreneurship on the progress and performance of sports performance entrepreneurial projects In order to study the impact of entrepreneurship on the entrepreneurial development and corporate performance of the sports performance industry, would you like to spare a few minutes to answer the following questions in this questionnaire carefully and authentically! We will promise you that your answers will be kept strictly confidential!

1. In your sports performance entrepreneurial project, which entrepreneurial spirits have an impact on the development of the project (multiple choices)
  A Knowledge literacy B Innovation spirit C Adventure spirit D Advance action E Achievement needs F Professional dedication H Both have a certain degree of influence
2. The current stage of your sports performance start-up industry is
  A start-up stage B growth stage C mature stage D decline stage
3. To what extent do you think the continuous development of new sports performance entrepreneurial projects will affect the development of enterprise performance
  A Very large B Average C Very little D Unfavorable
4. Do you think the sports performance business should continue to develop new products and businesses?
  A yes B no
5. How much do you think the spirit of risk-taking affects the progress of sports performance business
  A Very large B Average C Very little D Unfavorable
6. The degree to which you feel that achievement needs to be influenced by the progress of entrepreneurship in sports performance
  A Very large B Average C Very little D Unfavorable
7. How much do you think professionalism influences the progress of sports performance entrepreneurship
  A Very large B Average C Very little D Unfavorable
8. What do you think is the influence of the knowledge literacy of sports entrepreneurs and the progress of sports performance business
  A Very large B Average C Very little D Unfavorable
9. Do you think the degree of influence of advanced actions on the progress of sports performance business?
  A Very large B Average C Very little D Unfavorable
10. In your opinion, the extent to which the spirit of innovation has influenced the progress of sports performance entrepreneurship
  A Very large B Average C Very little D Unfavorable
  Thank you very much for your support and cooperation!
